# Need for Closure Moderates the Break in the Message Effect

**DOI:** 10.3389/fpsyg.2016.01879

**Published:** 2016-11-28

**Authors:** Dariusz Dolinski, Barbara Dolinska, Yoram Bar-Tal

**Affiliations:** ^1^Faculty of Psychology in Wroclaw, SWPS University of Social Sciences and HumanitiesWroclaw, Poland; ^2^Faculty of Social Sciences, Opole UniversityOpole, Poland; ^3^Sackler Faculty of Medicine, Tel Aviv UniversityTel Aviv, Israel

**Keywords:** mass-media, social influence, cognitive structuring, moral judgments

## Abstract

Cutting the message into smaller portions is a common practice in the media. Typically such messages consist of a headline followed by a story elaboration. In a series of studies Dolinski and Kofta (2001) have shown that such a break in the message increases the effect of the information provided in the headline over that of a story which actually contained information inconsistent with that headline. A possible explanation of this effect, based on the concept of the need for cognitive closure, is presented in the article. The experiment shows that break-in-the-message effect is found mainly for participants with high need for closure but not for those with low such need.

## Introduction

Cutting the message into smaller portions is a common practice in modern media all over the world. Typically, such reports consist of a headline (i.e., an announcement or a preview of the story) followed by the story elaboration (i.e., a detailed account of the event). The headline explains what happened. The event is briefly presented in a decontextualized manner, its most sensational aspects exposed. The story elaboration describes how this event transpired. Contextual knowledge is provided about the event’s circumstances, the actors’ social and psychological characteristics, and the story dynamics. Additionally, some experts in the field may present their opinions about the event. Importantly, the various parts of the story are usually separated in time. This happens most frequently on television and radio, where following presentations of the headline, the full story is presented with some delay. Temporal delays may also naturally occur when people read newspapers starting with a brief description of an event on the cover page and then later read the entire story inside the issue. On the Internet, the headline is often visible on the screen, but in order to obtain more detailed information one must scroll over it and click using a mouse. For example, under the headline “Serious danger in the supermarket” one may read on the cover page of a newspaper “Poisoned food has been found in one of the country’s most popular supermarkets. The situation seems to be very serious since more than 300 people visit this shop on a daily basis.” Subsequently (after the break) the reader finds the entire story inside the paper, which informs that a party of fresh milk (20 bottles) sold in this store yesterday contained a slightly higher amount of bacteria than is allowed according to sanitary regulations. The medical expert interviewed for the story says that these bacteria may lead to minor diarrhea in people who drink a lot of milk, but it is not particularly harmful to health. Store executives have initiated evaluation procedures, but it is still unclear why the milk supplier did carefully check product quality before shipping to the store. A detailed inspection is taking place in the dairy.

Dolinski and Kofta (2001) have experimentally demonstrated how splitting a news story affects recipients’ attributions of responsibility to the people presented in the story. The main manipulation in their research consisted of presenting a story as either an integrated, continuous message, or separating the headline from the elaboration by a break in time. The authors found that a break in the message increased perceived responsibility and moral disapproval of the person or persons implicated by the initial information presented about the story. Segmentation of a news story was also associated with more punitive judgments about these individuals (which was consistent with the initial headline but inconsistent with the subsequently elaborated message).

For example, in one of the experiments the participants were university students living in a dormitory who happened to listen that day to the university radio station. Approximately half of the dormitory rooms were set up so that the radio message was delivered in a continuous manner, while the other half of the rooms were presented with a message that was interrupted by a message that was itself interrupted by a piece of music, which is typical for most radio transmissions. (This procedure was made possible thanks to cooperation with the radio staff). Dormitory tenants were informed that a female student named Magda R. had reported to the police and testified that during a party she was raped by Marek W., who was on a short leave from military service. The participants in the continuous story condition then heard Marek W. describing this fateful night quite differently. In contrast, listeners in the break-in-the-message condition were informed that the story would be continued following a piece of rock music. Either immediately or after a delay the participants heard from Marek W, who explained that he had engaged in sex with Magda R., but that it was consensual. He also admitted that she used to be his girlfriend, and that they had a serious argument during the party. He stated his opinion that Magda R. accused him out of revenge. After the broadcast, experimenters knocked on students’ doors and asked them for their opinions about the incidents. Analysis of the results revealed that participants who had heard the story with the musical interruption were more certain that the accused had indeed raped the girl, attributed more responsibility to him, and made more punitive judgments than participants who heard the story without the interruption.

Although the effects of the structure of information have long been studied in psychology (e.g., Asch, 1946; Anderson, 1965), a more recent look at this phenomenon took into account the role of chunking of information. Petty et al. (2001) asked participants to read arguments for and against a proposed new exam policy. The arguments were presented in either a standard or chunked condition. The experiment showed that a stronger need for cognition amplifies the primacy effect when information is chunked. When the information was not chunked, this pattern was reversed: there were primacy effects for low need for cognition participants and recency effects for those with high need for cognition. Petty et al. (2001) explained their results by suggesting that high need for cognition individuals who are motivated to think process the initial chunk of information in greater depth, and therefore create an abstract of this information in the form of an attitude or schema. This cognitive structure may bias participants when encountering inconsistent information.

Dolinski and Kofta (2001) did not take into account individual differences, but they consistently obtained a primacy effect in their “break in the message” experiments. However, there is a substantial difference between these authors’ methodology and that employed by Petty et al. (2001) in that chunking does not imply breaking the message into two separate pieces with a time separation. It is the time gap which facilitates crystallization of the cognitive structure, which in turn leads to bias in the processing of the second segment, perhaps through confirmation bias. By confirmation bias we refer to the tendency to seek and interpret evidence in a manner congruent with existing beliefs, expectations or hypotheses (e.g., Koriat et al., 1980; Frey, 1981; Holton and Pyszczynski, 1989). In the headline phase the individual is informed that a dramatic event has happened. In the absence of contradicting information, at this stage the participant classifies the offender as a rapist. The information arriving at a later stage and suggesting the possibility that no rape occurred is ignored because of its incongruency with the cognitive structure which has already labeled the man a rapist. This explanation, however, implies heuristic thinking rather than the more careful thinking suggested by Petty et al. (2001). Both the categorization stage during the exposure to the headline phase (Thompson et al., 1994; Ford and Kruglanski, 1995), as well as confirmation bias (Jonas et al., 2001; Bar-Tal, 2010) more strongly indicate heuristic thinking than the vigilant thinking implied by the conceptualization suggested by Petty et al. (2001). Following this logic, in the present paper we examine the moderating effect of epistemic motivation to use heuristic thinking on the “breaking the news” phenomenon through the lens of the Need for Closure (NFC) concept described in Lay epistemology theory by Kruglanski (1989, 1990; Kruglanski and Webster, 1996). Lay epistemology theory suggests the presence of two stages in the knowledge acquisition process. In the first stage, the cognitive content (in lay epistemology terms, the hypothesis) is created. This stage is labeled as “seizing.” In the second stage this content is evaluated by reviewing it and comparing to other content the person is aware of. If it is not found to be inconsistent with other content, and there is no alternative explanation, the individual becomes certain that the content is valid. The length of the validation process depends on the epistemic mechanisms of “freezing” and “unfreezing.” Faster freezing implies less thorough examination, and therefore achieving certainty more quickly as to the validity of the hypothesized cognition. Unfreezing of the epistemic process implies a longer process of examination, examination of the consistency of a greater amount of content and of more possible alternative explanations. As long as the unfreezing stage is in progress, the individual cannot be certain about the validity of the cognitive content. The epistemic freezing–unfreezing mechanism is governed by epistemic motivation. The greatest attention in the theory is paid to the NFC. NFC refers to an individual’s desire for a definitive answer to a question and an aversion toward ambiguity. People with heightened NFC experience the absence of such an answer as aversive. According to Kruglanski, NFC is influenced by states (e.g., time pressure, conditions that render processing of information difficult, aversive, or laborious), while it also constitutes a more stable, trait-like characteristic. Cognitive processes used by high-NFC individuals to reduce uncertainty include avoiding inconsistent information and the use of category-based, non-systematic, and heuristic thinking. In other words, high-NFC individuals are characterized by more extensive and faster use of epistemic freezing. In contrast, individuals with low levels of NFC prefer to reduce uncertainty by using piecemeal or individuation processes (unfreezing)^1^. This preference is manifested in vigilant behavior that is based on a systematic and effortful search for relevant information, its evaluation, and its unbiased assimilation (Driscoll et al., 1991; Bar-Tal et al., 1997; Kruglanski et al., 2009). In terms of lay epistemology theory, the ‘breaking the message’ phenomenon is explained in the following way: when a headline is presented without an immediate following elaboration, it creates a schema or a hypothesis. Fast freezing of this content causes the individual to believe that it is accurate. Therefore, an encounter at a later stage with inconsistent information causes uncertainty and aversion. The avoidance of information inconsistent with the schema is characteristic of confirmation bias. When there is no break in the message, the headline is processed together with the rest of the information. The “seizing” stage will probably require more time, but it will result in content which is a combination of the headline and the subsequent information. Thus, it can be hypothesized that high-NFC individuals who tend to freeze faster and avoid schema-inconsistent information, in order to avoid the aversion caused by uncertainty, will be more affected by the ‘breaking the news’ phenomenon than low-NFC individuals. That is to say, an individual’s level of NFC moderates the effect of the phenomenon.

## Materials and Methods

### Participants

A group of 103 male university students aged 19 to 43 (*M* = 29.08; *SD* = 6.58) participated in the experiment in exchange for compensation of two tokens for lunch in the university cafeteria. Participants were randomly assigned to one of the conditions: control and experimental (break-in-the-message). Since only a few female students were available to us, we decided to have only male participants in the study.

### Procedure

Participants (in groups of 6 to 16 individuals) initially completed a scale designed to measure their NFC. We used the Polish version (Kossowska, 2003) of the NFC scale (Webster and Kruglanski, 1994). Respondents rated 32 items on a six-point scale (from 1 = completely disagree to 6 = completely agree). The reliability of the scale in the present study was Cronbach α = 0.75. The mean score for all items was calculated as *M* = 3.74; *SD* = 0.54. A higher mean score indicated a higher level of NFC.

Subsequently, they were asked to listen to part of a radio program “presenting a story about a young female student.” The story (based on one used by Dolinski and Kofta, 2001, study 1) started with the information that a 20-year-old student visited a local hospital, complaining of acute abdominal pain which had worsened over the preceding few days. The doctor on duty examined her briefly, diagnosed an ovarian inflammation, and admitted her to the hospital for further observation. In the early hours of the morning, however, the patient died. The autopsy showed a ruptured appendix. In the control condition, this part of the story was subsequently followed by more detailed information. In the break-in-the-message condition, however, at this moment a piece of music lasting about 2 min was played. The rest of the story was delivered after this break. The second part of the story (of course, exactly the same in both conditions) provided several justifications for the doctor’s actions. In brief, the participants were informed that his diagnosis was understandable owing to the fact that, in a routine examination, inflammation of the ovaries presents similar symptoms to that of appendicitis. In addition, during the doctor’s interview the patient stressed that she had been suffering from various gynecological diseases for several years. Additionally, the doctor could not operate immediately because the anaesthesiologist on duty in the hospital was busy at that moment. In both conditions participants were asked to answer three questions using a 7-point scale (ranging from 0 to 6):

(1) To what extent is the doctor on duty responsible for the patient’s death (from 0 “not at all responsible” to 6 “fully responsible”)
(2) The prosecutor demands a sentence of 6 years in prison for the doctor, while his lawyer demands an acquittal. The judge can acquit him or order from 1 to 6 years in prison. What would be a fair judgment? (from 0 “acquitted of charges” to 6 “six years in prison”)
(3) What is your moral evaluation of this doctor? (from 0 “very positive” to 6 “very negative”)

These items as described created a unidimensional measure of moral disapproval. (Cronbach’s α = 0.91; *M* = 2.51; *SD* = 1.46).

All participants gave their written informed consent to take part in the study and were fully debriefed after completion of the experiment. The design and the experiment conditions for the study were approved by the local University of Social Science and Humanities Ethics Committee in accordance with the Helsinki Declaration.

## Results and Discussion

To test the hypothesis we performed a hierarchical regression in two steps. In the first step, we examined the two main effects [manipulation of the time break as a dummy variable, and NFC score; *F*_(2,100)_ = 23.47, *p* < 0.01]. In the second step we introduced the interaction effect [*F*_(3,99)_ = 22.20, *p* < 0.01]. Total explain variance was 0.40. **Table 1** presents the results of our analysis.

**Table 1:** Regression analysis.

	*B*	*SE*	β	*T*	Δ*R*^2^	*F* Change	Low CI for *B*	High CI for *B*
Step 1					0.32	23.47^∗∗^		
Manipulation	1.38	0.24	0.47	5.74^∗^			0.90	1.86
Need for Closure (NFC)	0.41	0.12	0.28	3.40^∗^			0.17	0.65
Step 2					0.08	13.70^∗∗^		
Manipulation X NFC	0.90	0.24	0.50	3.70^∗^			0.42	1.38

*^∗^*p* < 0.05; ^∗∗^*p* < 0.01.*

The table shows that all three effects are significant. As in Dolinski and Kofta’s (2001) experiments we have shown that separating the headline from the elaborated part by inserting a time break increased the severity of negative moral judgments about the accused person. This finding clearly suggests that it is not only the content of the message that is important in social influence, but also the manner in which the news is delivered. As widespread practices in modern mass media, segmentation and time separation in the delivery of messages may seriously influence the opinions of the audience. In addition, our research has shown a relation between the need for cognitive closure and the level of moral disapproval. High NFC was connected with more punitive assessments. This effect may be explained by greater rigidity among high-NFC individuals. Kossowska and Van Hiel (2003) demonstrated that NFC is associated with intolerance of those who violate conventional norms (see also: Kosic et al., 2004; Van Hiel et al., 2004). For interpretation of the interaction we examined the simple slopes of the dependent measure on the manipulation of breaking up the news for individuals with high and low NFC levels (1 SD above and below the mean) Using Hayes Process Model 1 (2013). The results shows that while for low NFC individuals there a non-significant effect of the manipulation of the time break (*b* = 0.50, *SE*, = 0.33, *t* = 1.52, *p* = ns, LLCI = -0.15, ULCI = 1.15), this effect is significant in the case of high-NFC individuals (*b* = 1.39, *SE* = 0.22, *t* = 6.17, *p* < 0.01, LLCI = 0.95, ULCI = 1.85).

From the practical point of view, one may therefore conclude that the media practice of cutting the message into two portions is especially (or perhaps exclusively) dangerous for high-NFC recipients. If media producers would like to avoid leading such people to premature, overconfident moral judgements, they should limit the headline portion of the message to a brief summary or, on the contrary, to present it in rather elaborated form, instead of utilizing the intermediate-length presentation prevalent today.

We are aware that this study should be treated only as a first step in investigating the role played by NFC in the break-in-the-message phenomenon. Although Dolinski and Kofta (2001) have shown that this effect is quite robust, since it emerged on different measures, in different samples, and in different experimental conditions, the moderating role of NFC should be investigated further. For example, it is particularly interesting to see whether the pattern of results would be the same if participants were initially informed about the power of the break-in-the-message effect and/or were encouraged to rely on the information presented in the second phase. Another set of questions concerns the valence of the story presented to participants. In both experiments by Dolinski and Kofta (2001) and in this paper, threatening and negative acts were presented. The question arises of whether the pattern of results would be the same if a neutral or laudable act were presented in the headline. Yet another problem is connected with the information delivered in the second phase. While it has been demonstrated that the break-in-the-message effect occurs when this information is ambiguous and/or uncertain, it is an open question whether the same would be observed if the second part clearly contradicted the first one.

## Ethics Statement

Ethics Committee of SWPS University, Wroclaw Faculty Informed consent was obtained from all individual participants included in the study.

## Author Contributions

DD substantially contributed to the conception of the work, interpretation of data for the work, drafting the work, and final approval of the version to be published; and also the agreement to be accountable for all aspects of the work in ensuring that questions related to the accuracy or integrity of any part of the work are appropriately investigated and resolved. BD substantially contributed to the design of the work, interpretation of data for the work, revisiting the work critically for important intellectual content; and final approval of the version to be published; and also agreement to be accountable for all aspects of the work in ensuring that questions related to the accuracy or integrity of any part of the work are appropriately investigated and resolved. YB-T substantially contributed to the design of the work, data analysis, and interpretation of data for the work, drafting the work; and final approval of the version to be published; and also agreement to be accountable for all aspects of the work in ensuring that questions related to the accuracy or integrity of any part of the work are appropriately investigated and resolved.

## Conflict of Interest Statement

The authors declare that the research was conducted in the absence of any commercial or financial relationships that could be construed as a potential conflict of interest.

## Footnotes

1 It should be noted that while NFC is very widely used in social psychology, there is a variety of concepts with different theoretical backgrounds but similar predictions applied in psychological research. For example: need for structure, tolerance for ambiguity, open and closed mind, personal need for structure, etc (Frenkel Brunswik, 1949; Rokeach, 1960; Bunder, 1962; Kagan, 1972; Epstein, 1991; Neuberg and Newsom, 1993). Although the various theoretical elaborations differ from one another, there is broad agreement among them regarding the cognitive processes used by high-NFC and low-NFC individuals to reduce uncertainty.
